# Anticoagulant Treatment Regimens in Patients With Covid‐19: A Meta‐Analysis

**DOI:** 10.1002/cpt.2504

**Published:** 2021-12-19

**Authors:** Anselm Jorda, Jolanta M. Siller‐Matula, Markus Zeitlinger, Bernd Jilma, Georg Gelbenegger

**Affiliations:** ^1^ Department of Clinical Pharmacology Medical University of Vienna Vienna Austria; ^2^ Division of Cardiology Department of Medicine II Medical University of Vienna Vienna Austria; ^3^ Center for Preclinical Research and Technology CEPT Department of Experimental and Clinical Pharmacology Medical University of Warsaw Warsaw Poland

## Abstract

Coronavirus disease 2019 (COVID‐19) is associated with a hypercoagulable state. It has been hypothesized that higher‐dose anticoagulation, including therapeutic‐dose and intermediate‐dose anticoagulation, is superior to prophylactic‐dose anticoagulation in the treatment of COVID‐19. This meta‐analysis evaluated the efficacy and safety of higher‐dose anticoagulation compared with prophylactic‐dose anticoagulation in patients with COVID‐19. Ten randomized controlled open‐label trials with a total of 5,753 patients were included. The risk of death and net adverse clinical events (including death, thromboembolic events, and major bleeding) were similar between higher‐dose and prophylactic‐dose anticoagulation (risk ratio (RR) 0.96, 95% CI, 0.79–1.16, *P* = 0.66 and RR 0.87, 95% CI, 0.73–1.03, *P* = 0.11, respectively). Higher‐dose anticoagulation, compared with prophylactic‐dose anticoagulation, decreased the risk of thromboembolic events (RR 0.63, 95% CI, 0.47–0.84, *P* = 0.002) but increased the risk of major bleeding (RR 1.76, 95% CI, 1.19–2.62, *P* = 0.005). The risk of death showed no statistically significant difference between higher‐dose anticoagulation and prophylactic‐dose anticoagulation in noncritically ill patients (RR 0.87, 95% CI, 0.50–1.52, *P* = 0.62) and in critically ill patients with COVID‐19 (RR 1.04, 95% CI, 0.93–1.17, *P* = 0.5). The risk of death was similar between therapeutic‐dose vs. prophylactic‐dose anticoagulation (RR 0.92, 95% CI 0.69–1.21, *P* = 0.54) and between intermediate‐dose vs. prophylactic‐dose anticoagulation (RR 1.01, 95% CI 0.63–1.61, *P* = 0.98). In patients with markedly increased d‐dimer levels, higher‐dose anticoagulation was also not associated with a decreased risk of death as compared with prophylactic‐dose anticoagulation (RR 0.86, 95% CI, 0.64–1.16, *P* = 0.34). Without any clear evidence of survival benefit, these findings do not support the routine use of therapeutic‐dose or intermediate‐dose anticoagulation in critically or noncritically ill patients with COVID‐19.

**Study Highlights**

**WHAT IS THE CURRENT KNOWLEDGE ON THE TOPIC?**

☑ Current guidelines recommend routine venous thromboembolism prophylaxis with prophylactic‐dose low molecular weight heparin in patients hospitalized with coronavirus disease 2019 (COVID‐19). The clinical role of higher‐dose anticoagulation remains unknown.

**WHAT QUESTION DID THIS STUDY ADDRESS?**

☑ This meta‐analysis aimed to assess the efficacy and safety of higher‐dose anticoagulation compared with prophylactic‐dose anticoagulation.

**WHAT DOES THIS STUDY ADD TO OUR KNOWLEDGE?**

☑ There is currently insufficient evidence of survival benefit of therapeutic‐dose or intermediate‐dose anticoagulation compared with prophylactic‐dose anticoagulation in noncritically ill and in critically ill patients hospitalized with COVID‐19. Further, this analysis does not support the routine use of d‐dimer as an isolated biomarker to guide anticoagulation dose escalation.

**HOW MIGHT THIS CHANGE CLINICAL PHARMA‐COLOGY OR TRANSLATIONAL SCIENCE?**

☑ Our findings do not support the routine use of therapeutic‐dose or intermediate‐dose anticoagulation. Patients hospitalized with COVID‐19 should continue to receive prophylactic‐dose anticoagulation according to current guidelines.

Coronavirus disease 2019 (COVID‐19) is associated with severe inflammation and organ damage.^1^ COVID‐19 also shows significant prothrombotic activity leading to widespread thrombosis and microangiopathy.^2^, ^3^, ^4^ Additional factors, such as endothelial injury, microvascular inflammation, enhanced complement activation, and elevated plasma coagulation factors, might contribute to a hypercoagulable state.^5^, ^6^, ^7^ The ensuing coagulopathy can result in macrothrombosis, such as pulmonary embolism, but may also lead to microthrombosis, which is mostly left undiagnosed.^2^ Hence, anticoagulation (widely performed with low molecular weight heparin (LMWH)) has been identified as a beneficial treatment strategy in patients with COVID‐19 and is associated with improved survival.^8^, ^9^ Although current guidelines recommend venous thromboembolism prophylaxis with LMWH in a prophylactic dose regimen, the role of intermediate and even therapeutic doses remains unknown. Given its antithrombotic, anti‐inflammatory, and possibly antiviral properties,^10^, ^11^, ^12^ higher doses of LMWH for venous thromboprophylaxis have been hypothesized to improve clinical outcomes. Observational data suggested that both therapeutic‐dose and prophylactic‐dose anticoagulation might be associated with lower in‐hospital mortality compared with no anticoagulation.^13^ D‐dimer levels were identified to be associated with vascular thrombosis and a poor clinical outcome,^14^, ^15^ which might suggest a strategy of d‐dimer‐guided anticoagulation. Although some retrospective observational data shows anticoagulation beyond prophylactic doses to be associated with reduced mortality,^16^ opposing data suggests an increased risk of bleeding.^17^ The optimal dosing of anticoagulation has been the subject of several randomized clinical trials (RCTs). This meta‐analysis aims to compare clinical outcomes associated with higher‐dose and prophylactic‐dose anticoagulation in patients hospitalized with COVID‐19.

## METHODS

### Data sources and searches

This meta‐analysis was registered at PROSPERO under the registration number CRD42021278098. We conducted a systematic search in Medline, Embase, Web of Science, and the preprint server medRxiv from database inception through the final search date of November 24, 2021. We used the predefined search terms: (COVID‐19 OR coronavirus disease 2019 OR severe acute respiratory syndrome‐coronavirus 2 (SARS‐CoV‐2)) AND (heparin OR enoxaparin OR anticoagulation). After checking for clinical trials, relevant articles were screened based on their title. Next, articles were assessed for their eligibility by reading the abstract and, if necessary, the full text. No language, publication date, or publication status restrictions were applied. References of identified articles and previous meta‐analyses were searched for additional literature.

### Study selection

This meta‐analysis was prepared in accordance with the Preferred Reporting Items for Systematic Reviews and Meta‐Analyses (PRISMA) statement (**Table** **S1**) and performed according to established methods, as described previously.^18^ Only full‐text articles were included. We included trials that (i) were RCTs, (ii) compared at least two dosing regimens of anticoagulation, and (iii) reported at least one of our outcomes of interest (all‐cause death, major bleeding, thromboembolic events, venous thromboembolic events, and arterial thromboembolic events). Ongoing, retrospective, other non‐RCT, and duplicate studies were excluded. Because observational data are inherently subject to various types of bias, we limited the study selection to RCTs, which are the gold standard for creating strong clinical evidence about the efficacy and safety of an intervention. Studies were excluded if one could determine, from the title, abstract, or both, that the study did not meet the inclusion criteria. The full text of a study in question was acquired and evaluated if an article could not be excluded with certainty. The comprehensive literature search and study selection were independently carried out by two reviewers (authors A.J. and G.G.). Any discrepancies were resolved after personal discussion and consensus.

### Data extraction and quality assessment

Three dosing strategies of anticoagulation are described throughout this article: (i) therapeutic‐dose anticoagulation (mostly 1 mg/kg of enoxaparin twice daily or rivaroxaban 20 mg once daily); (ii) intermediate dose (mostly 1 mg/kg of enoxaparin once daily); and (iii) prophylactic dose (mostly 40 mg of enoxaparin once daily). Therapeutic‐dose and intermediate‐dose are summarized as “higher‐dose anticoagulation.” In case other LMWHs (bemiparin, dalteparin, or tinzaparin), fondaparinux, or unfractionated heparin were used, an equivalent therapeutic, intermediate, or prophylactic dose was administered.

The primary efficacy outcome was death from any cause. The outcomes were assessed at day 21 (ATTACC and REMAP‐CAP), day 28 (HESACOVID and RAPID), or day 30 (ACTION, INSPIRATION, BEMICOP, HEP‐COVID, XCOVID‐19, and Perepu *et al*.) after randomization. Two trials (ATTACC and REMAP‐CAP) reported only on in‐hospital deaths. The secondary efficacy outcome was thromboembolic events, which include arterial thromboembolic events (i.e., stroke, myocardial infarctions, peripheral arterial thromboembolisms, and others) and venous thromboembolic events (i.e., deep venous thrombosis, pulmonary embolism, and others). Whenever possible, we reported both the composite outcomes and the subtypes of thromboembolic events. We also calculated the composite of net adverse clinical events (NACE: a composite of death, venous thromboembolism, and major bleeding). The primary safety outcome was major bleeding. Major bleeding event definitions differed between trials and were defined according to the International Society on Thrombosis and Hemostasis (ISTH) guidelines,^19^ the Bleeding Academic Research Consortium (BARC) type 3 or 5, and the Thrombolysis in Myocardial Infarction (TIMI) bleeding criteria.^20^ The frequency of any bleedings, which includes major and non‐major bleedings, was also assessed. Due to the variable study designs of the included trials, the pooling of other relevant end points (need for intubation, need for extracorporeal membrane oxygenation, duration of hospitalization, duration of intensive care unit stay, or others) was not feasible.

We performed predefined subgroup analyses for multiple end points comparing (i) critically ill vs. noncritically ill patients, (ii) prophylactic‐dose vs. intermediate‐dose anticoagulation, (iii) prophylactic vs. therapeutic dosing, and (iv) patients with markedly increased d‐dimer levels. Due to the high heterogeneity of the studies, the highest d‐dimer level group of each study was used for this subgroup analysis. High d‐dimer levels were defined as a two‐fold increase of the upper limit of normal (ATTAC and RAPID), a four‐fold increase of the upper limit of normal (HEP‐COVID), or as plasma levels over 500 ng/mL (BEMICOP) or 1000 ng/mL (INSPIRATION). Sensitivity analyses were performed by removing each singular trial from the overall analyses and testing the impact of fixed vs. random‐effect models of each outcome. All reports eligible for analysis were assessed using the Cochrane Risk of Bias Tool (**Table** **S2**). Publication bias was assessed by preparing funnel plots based on fixed‐effect models of the key outcomes of this meta‐analysis and visually inspecting them (**Figure** **S1** **A**–**F**). Funnel plots did not show obvious asymmetry, indicating no clear evidence of publication bias.

### Data synthesis and analysis

The variables of the defined outcome parameters were extracted from full‐text publications and, if available, supplementary documents. Categorical variables are reported as frequencies and percentages. Results were pooled according to the inverse variance model. Risk ratios (RRs) with 95% confidence intervals (95% CIs) of each study and of the pooled data are reported. Unadjusted *P* values are reported throughout, with hypothesis testing set at the 2‐tailed significance level of below 0.05. Statistical heterogeneity and homogeneity between studies were assessed by inconsistency testing (*I* ^2^). Percentages lower than 25% (*I* ^2^ = 25), 50% (*I* ^2^ = 50), and 75% (*I* ^2^ = 75) correspond to low, medium, and high heterogeneity, respectively.^21^ Despite the observed low to moderate statistical heterogeneity, we used a random‐effects model because of methodological and clinical differences between the included trials and intention to generalize the findings beyond the included studies.^22^, ^23^ The statistical analysis was carried out using Review Manager (version 5.4. Copenhagen: The Nordic Cochrane Centre, The Cochrane Collaboration, 2014).

## RESULTS

### Description of studies

Our initial literature search identified 10,208 articles. After removal of duplicates and screening based on article type and title, 19 articles were assessed for eligibility. Nine articles were excluded because they were protocols (*n* = 4), retrospective studies (*n* = 3), or correspondence (*n* = 2). Ten studies with a total of 5,753 patients with COVID‐19 were included in the final analysis.^24^, ^25^, ^26^, ^27^, ^28^, ^29^, ^30^, ^31^, ^32^, ^33^ More information on the literature search is provided in the flow diagram (**Figure** **S2**). **Table** **1** summarizes the study design of the 10 included trials (further information in **Table** **S3** and **S4**). Nine of the 10 studies were open‐label, RCTs; only in the HEP‐COVID trial, patients and investigators were blinded to treatment assignment.^33^ Three trials included only critically ill patients (HESACOVID, INSPIRATION, and RAMP‐CAP), 3 trials included both (ACTION, HEP‐COVID, Perepu *et al*.), and 4 trials included only noncritically ill patients (ATTAC, BEMICOP, RAPID, and XCOVID‐19). Seven of the 10 trials compared therapeutic‐dose with prophylactic‐dose anticoagulation, and 3 trials compared intermediate‐dose with prophylactic‐dose anticoagulation. One trial (XCOVID‐19) was not peer‐reviewed at the time of the literature search and was retrieved from the preprint server medRxiv.^30^ Of note, the ACTION trial used rivaroxaban instead of an LMWH as the preferred anticoagulant agent. Dual antiplatelet therapy was an exclusion criterion in four trials (ATTACC, RAPID, HEP‐COVID, and REMAP‐CAP trials). Two trials (ACTION and BEMICOP) also excluded patients receiving an antiplatelet monotherapy.

**Table 1:** Overview of included trials

Study	Design	Study population	Median days from symptom onset to randomization	Follow‐up, days	Dose of anticoagulation^a^ (enoxaparin or rivaroxabzn)	Patients included
ACTION, 2021^28^	Open‐label RCT	Hospitalized with COVID‐19 (stable and unstable)	10	30	Therapeutic: 1 mg/kg twice daily OR rivaroxaban 20 mg twice daily vs. prophylactic: 40 mg once daily	615 (311 vs. 304)
ATTAC, 2021^24^	Open‐label RCT	Hospitalized with COVID‐19 (noncritically ill)	Not reported	21	Therapeutic: 1 mg/kg twice daily vs. prophylactic: 40 mg once daily	2,219 (1,171 vs. 1,048)
BEMICOP, 2021^29^	Open‐label RCT	Hospitalized with COVID‐19 (noncritically ill)	8	30	Therapeutic: 115 IU/kg once daily^b^ vs. prophylactic: 3,500 IU once daily^b^	65 (32 vs. 33)
HEP‐COVID, 2021^33^	Double‐blind RCT	Hospitalized with COVID‐19 (noncritically and critically ill)	Not reported	30	Therapeutic: 1 mg/kg twice daily vs. prophylactic: 30–40 mg once daily	253 (129 vs. 124)
HESACOVID, 2020^27^	Open‐label RCT	Hospitalized with COVID‐19 (and ARDS)	Not reported	28	Therapeutic: 1 mg/kg twice daily vs. prophylactic: 40 mg once daily	20 (10 vs. 10)
INSPIRATION, 2021^25^	Open‐label RCT	Admitted to ICU with COVID‐19	11	30	Intermediate: 1 mg/kg once daily vs. prophylactic: 40 mg once daily	562 (276 vs. 286)
Perepu *et al*., 2021^31^	Open‐label RCT	Hospitalized with COVID‐19 (ICU and/or coagulopathy)	Not reported	30	Intermediate: 1 mg/kg once daily vs. prophylactic: 40 mg once daily	173 (87 vs. 86)
RAPID, 2021^32^	Open‐label RCT	Hospitalized with COVID‐19 (and elevated d‐dimer)	7^c^	28	Therapeutic: 1 mg/kg twice daily vs. prophylactic: 40 mg once daily	465 (228 vs. 237)
REMAP‐CAP, 2021^26^	Open‐label RCT	Hospitalized with COVID‐19 (critically ill)	not reported	21	Therapeutic: 1 mg/kg twice daily vs. prophylactic: 40 mg once daily	1,098 (534 vs. 564)
XCOVID‐19, 2021^d^ ^30^	Open‐label RCT	Hospitalized with COVID‐19 (noncritically ill)	6–7	30	Intermediate: 40 mg twice daily vs. prophylactic: 40 mg once daily	183 (91 vs. 92)

ARDS, acute respiratory distress syndrome; COVID‐19, coronavirus disease 2019; ICU, intensive care unit; RCT, randomized controlled trial.

^a^ Anticoagulation with bemiparin.

^b^ Possible changes according to local practice and modification according to body weight and kidney function. Other anticoagulant agents, including other low molecular weight heparins, fondaparinux, or unfractionated heparin, were only infrequently used.

^c^ Mean.

^d^ Non‐peer‐reviewed study (preprint accessed via medrxiv.org).

### Primary efficacy outcome: Death

**Figure 1:**
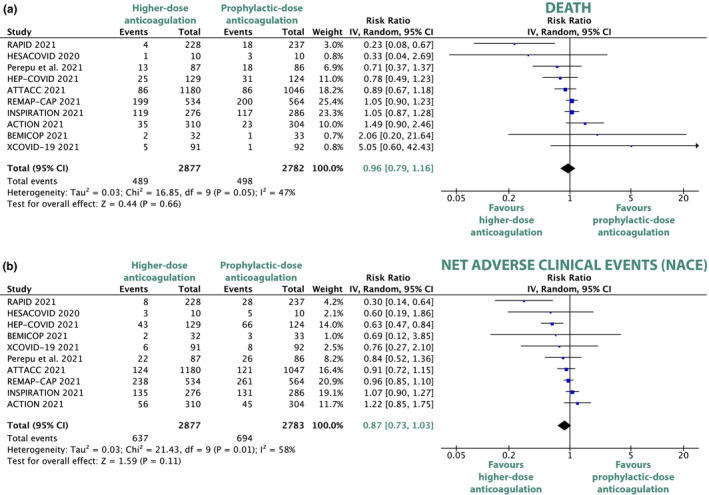
Forest plots depicting risk ratios of all‐cause death (**a**) and net adverse clinical events (**b**) for comparison between higher‐dose and prophylactic‐dose anticoagulation. Net adverse clinical events are a composite of all‐cause death, venous thromboembolism, and major bleeding. CI, confidence interval; COVID, coronavirus disease. [Colour figure can be viewed at wileyonlinelibrary.com]

All 10 included trials reported on death. In the overall analysis, there was no statistically significant difference in the relative risk of death between higher‐dose and prophylactic‐dose anticoagulation (RR 0.96, 95% CI, 0.79–1.16, *P* = 0.66; **Figure** **1a**). The absolute risk reduction, relative risk reduction (RRR), and number needed to treat or harm of the major outcomes of this study are shown in **Table** **2**.

**Table 2:** The absolute and relative risk reduction and number needed to treat/harm for higher‐dose as compared with prophylactic‐dose anticoagulation

	Absolute risk: higher dose, %	Absolute risk: prophylactic dose, %	Absolute risk reduction, %	Risk ratio	Relative risk reduction, %	Number needed to treat	Number needed to harm	*P* value
Death	17.0	17.9	0.90	0.96	4.0	111	na	0.66
Thromboembolic events	4.18	7.13	2.95	0.63	37.0	35	na	**0.002**
Major bleeding events	2.44	1.40	−1.03	1.76	−76.0	na	97	**0.005**

*P* values below 0.05 were considered statistically significant and are shown in bold.

### Secondary efficacy outcomes

In the net clinical benefit analysis (NACE), there was no statistically significant difference between the higher‐dose and the prophylactic‐dose anticoagulation group (RR 0.87, 95% CI, 0.73–1.03, *P* = 0.11; **Figure** **1b**).

Higher‐dose anticoagulation resulted in a statistically significant 37% RRR of the composite of thromboembolic events (RR 0.63, 95% CI, 0.47–0.84, *P* = 0.002; **Figure** **S3**) compared with prophylactic‐dose anticoagulation.

**Figure 2:**
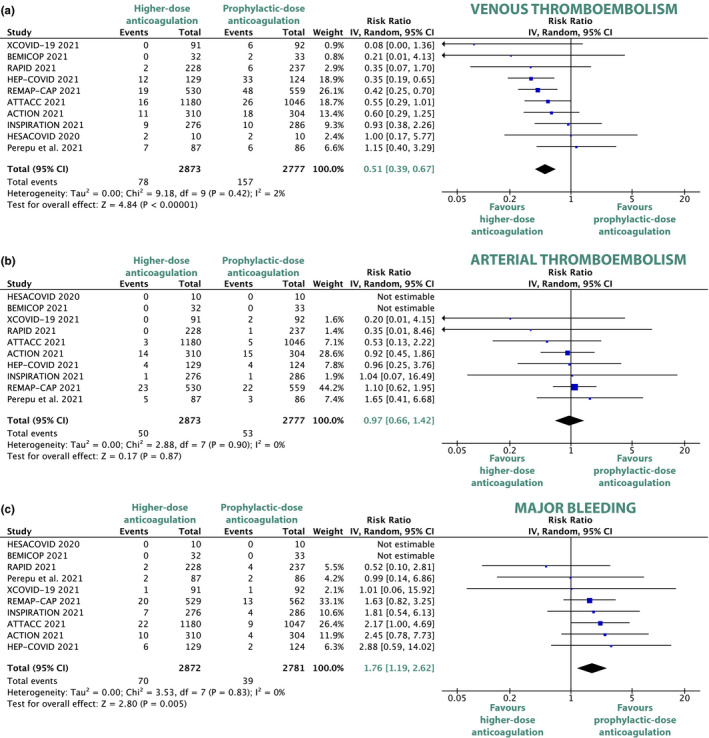
Forest plots depicting risk ratios of venous thromboembolism (**a**), arterial thromboembolism (**b**), and major bleeding (**c**) for comparison between higher‐dose and prophylactic‐dose anticoagulation. CI, confidence interval; COVID, coronavirus disease. [Colour figure can be viewed at wileyonlinelibrary.com]

Even more so, higher‐dose anticoagulation was associated with a significant 49% RRR of venous thromboembolic events (RR 0.51, 95% CI, 0.39–0.67, *P* < 0.00001; **Figure** **2a**) compared with prophylactic‐dose anticoagulation. Pulmonary embolisms also occurred significantly less frequently with higher‐dose anticoagulation as compared with prophylactic‐dose anticoagulation (RR 0.38, 95 95% CI, 0.26–0.55, *P* < 0.00001; **Figure** **S4**).

No statistically significant difference of arterial thromboembolic events was detected between higher‐dose and prophylactic‐dose anticoagulation (RR 0.97, 95% CI, 0.66–1.42, *P* = 0.87; **Figure** **2b**). The arterial thromboembolism subtypes of stroke (RR 0.85, 95% CI, 0.40–1.82, *P* = 0.68; **Figure** **S5**), myocardial infarction (RR 0.74, 95% CI, 0.44–1.25, *P* = 0.27; **Figure** **S6**), and peripheral arterial thromboembolism (RR 1.63, 95% CI, 0.54–4.93, *P* = 0.38; **Figure** **S7**) did not differ significantly between the higher‐dose and the prophylactic‐dose groups.

### Primary safety outcome: Major bleeding

All 10 included trials reported on major bleeding. Bleeding definitions were heterogeneous between trials (3 distinct bleeding definitions BARC, TIMI, and ISTH were used). Compared with prophylactic‐dose anticoagulation, higher‐dose anticoagulation was associated with a significantly increased risk of major bleedings (RR 1.76, 95% CI, 1.19–2.62, *P* = 0.005; **Figure** **2c**) and any bleedings (RR 2.08, 95% CI, 1.12, 3.85, *P* = 0.02; **Figure** **S8**).

### Subgroup analysis: Noncritically and critically ill patients

**Figure 3:**
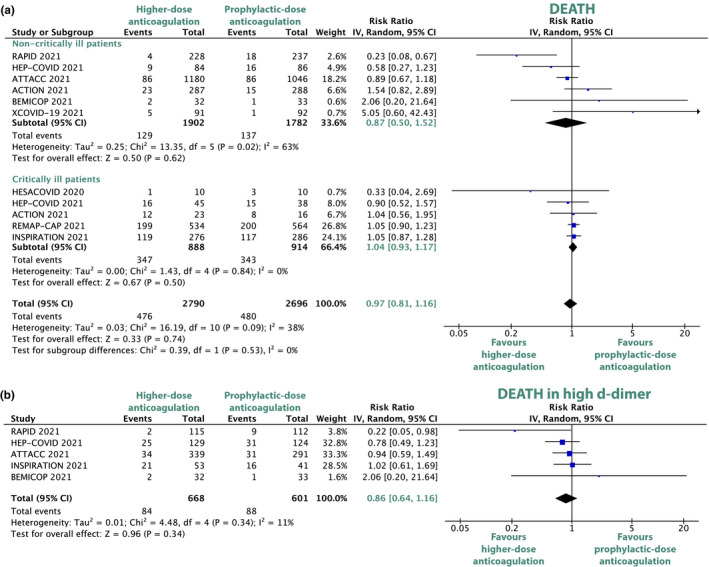
Forest plots depicting risk ratios of all‐cause death in critically ill and noncritically ill patients (**a**) and death in patients with high d‐dimer (**b**). The trial by Perepu *et al*. included in but both critically and noncritically ill patients but did not report on all‐cause death in the respective subgroups. CI, confidence interval; COVID, coronavirus disease. [Colour figure can be viewed at wileyonlinelibrary.com]

The risk of all‐cause death did not differ significantly with higher‐dose anticoagulation compared with prophylactic‐dose anticoagulation in noncritically ill (RR 0.87, 95% CI, 0.50–1.52, *P* = 0.38; **Figure** **3a**) and in critically ill patients with COVID‐19 (RR 1.04, 95% CI, 0.93–1.17, *P* = 0.5; **Figure** **3a**). As compared with prophylactic‐dose anticoagulation, higher‐dose anticoagulation was associated with a statistically significant decrease in thromboembolic events in noncritically ill (RR 0.39, 95% CI, 0.25–0.61, *P* < 0.0001; **Figure** **S9**) and in critically ill patients (RR 0.67, 95% CI, 0.49–0.93, *P* = 0.02; **Figure** **S9**). In both noncritically and critically ill patients with COVID‐19, major bleeding was numerically increased with high‐dose anticoagulation but did not become statistically significant (RR 1.53, 95% CI, 0.82–2.85, *P* = 0.19 and RR 1.78, 95% CI, 0.99–3.21, *P* = 0.05, respectively; **Figure** **S10**).

### Subgroup analysis: Therapeutic‐dose and intermediate‐dose vs. prophylactic‐dose anticoagulation

#### Therapeutic‐dose vs. prophylactic‐dose anticoagulation

The primary outcome of death did not differ statistically significantly between therapeutic‐dose and prophylactic‐dose anticoagulation (RR 0.92, 95% CI 0.69–1.21, *P* = 0.54; **Figure** **S11**). Treatment with therapeutic‐dose anticoagulation decreased the relative risk for thromboembolic events by 42% (RR 0.58, 95% CI, 0.45–0.73, *P* < 0.0001; **Figure** **S12**) and increased the relative risk of major bleeding events by 83% (RR 1.83, 95% CI, 1.19–2.83, *P* = 0.006; **Figure** **S13**) compared with prophylactic‐dose anticoagulation.

#### Intermediate‐dose vs. prophylactic‐dose anticoagulation

The comparison of intermediate‐dose with prophylactic‐dose anticoagulation showed no statistically significant difference in the risk of death (RR 1.01, 95% CI 0.63–1.61, *P* = 0.98; **Figure** **S14**). The effect of intermediate‐dose and prophylactic‐dose anticoagulation on the risk of thromboembolic events and major bleedings also did not differ (RR 0.84, 95% CI, 0.32–2.19, *P* = 0.72 and RR 1.45, 95% CI, 0.55–3.81, *P* = 0.45, respectively; **Figure** **S15,** **Figure** **S16**).

### Subgroup analysis: High d‐dimer levels

Five trials (ATTACC, INSPIRATION, BEMICOP, RAPID, and HEP‐COVID) reported the outcome of death for the subgroup of patients with markedly increased d‐dimer levels. In patients with high d‐dimer levels, higher‐dose anticoagulation was not associated with a decreased risk of death compared with prophylactic‐dose anticoagulation (RR 0.86, 95% CI, 0.64–1.16, *P* = 0.34; **Figure** **3b**).

### Sensitivity analyses

Sensitivity analyses for the random‐effect model and a fixed‐effect model showed no significant change in the overall and subgroup results (i.e., no result changed from statistically significant to not statistically significant or vice versa). The sequential exclusion of every single study had no considerable impact on the key outcomes of death, major bleeding, and the thromboembolic outcomes (i.e., venous thromboembolisms, arterial thromboembolisms, and their composite). The exclusion of the non‐peer‐reviewed XCOVID‐19 trial had no impact on the main findings. After exclusion of the ACTION trial, the only trial that primarily used rivaroxaban instead of LMWH, the risk reduction of death in the subgroup of noncritically ill patients remained statistically nonsignificant (RR 0.73, 95% CI, 0.37–1.45, *P* = 0.37; **Figure** **S17**). There was no statistically significant subgroup difference in the risk of death between noncritically and critically ill patients (χ^2 ^= 0.39, *P* = 0.53; **Figure** **3a**).

## DISCUSSION

This meta‐analysis comprises 10 trials (over 5,000 patients) comparing higher‐dose with prophylactic‐dose anticoagulation in patients hospitalized with COVID‐19.

**Figure 4:**
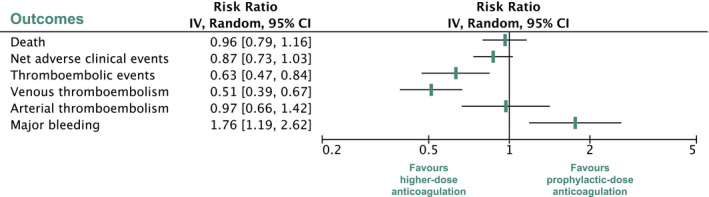
Summary of key outcomes of the overall analysis. Net adverse clinical events are a composite of death, venous thromboembolism, and major bleeding. CI, confidence interval. [Colour figure can be viewed at wileyonlinelibrary.com]

In the overall study population, higher‐dose anticoagulation was not associated with lower mortality compared with prophylactic‐dose anticoagulation. This key finding is consistent with current guidelines, which do not recommend routine administration of greater than prophylactic doses of anticoagulation in patients hospitalized with COVID‐19.^34^, ^35^ Similarly, a recently published meta‐analysis including seven trials showed no survival benefit with higher‐dose anticoagulation.^36^ Our analyses included 3 additional trials and could confirm previous findings. Higher‐dose anticoagulation decreased the risk of venous thromboembolism and increased the risk of major bleeding to a similar extent. These counteracting effects appear to cancel each other out in terms of all‐cause death. **Figure** **4** summarizes the main outcomes of this study. Of note, these results are only applicable for a population that largely excluded patients receiving dual antiplatelet therapy or patients with increased risk of bleeding. All trials except for the HESACOVID and XCOVID‐19 trials explicitly excluded patients with a low platelet count (< 50,000 or < 100,000/µL). The net benefit of higher‐dose anticoagulation in these relevant groups of patients remains unknown.

Rivaroxaban was used as the preferred anticoagulant agent in the ACTION trial, which showed a numerically higher risk of death with therapeutic‐dose anticoagulation compared with prophylactic anticoagulation (**Figure** **1**). Rivaroxaban is a small molecule with a distribution volume exceeding that of heparin,^37^, ^38^ potentially allowing it to better access lung tissue and prevent alveolar thrombosis. Further, rivaroxaban is easier to use in the outpatient setting because of its oral administration but is therefore mostly limited to noncritically ill patients. However, it does not share the same pleiotropic effects of heparin, which have been postulated to add to the benefits of heparin. It is unclear whether this finding reflects a clinical difference between anticoagulant treatment with rivaroxaban and LMWH in patients with COVID‐19.

In contrast to the seven trials comparing therapeutic‐dose with prophylactic‐dose anticoagulation, the three trials investigating intermediate‐dose enoxaparin (INSPIRATION, XCOVID‐19, and Perepu *et al*.) showed similar risks of thromboembolic events and major bleeding between intermediate‐dose and prophylactic‐dose anticoagulation. Consequently, intermediate‐dose anticoagulation may not be as effective as therapeutic‐dose anticoagulation in preventing COVID‐19‐associated thrombosis. Notably, in the REMAP‐CAP and the ATTACC trials, over 50% and over 26% of the prophylactic‐dose group received treatment with intermediate‐dose anticoagulation, respectively.

Time of initiation of anticoagulant treatment might play a role in the management of patients with COVID‐19. The average time from symptom onset to randomization was between 6 and 11 days. In patients hospitalized with COVID‐19, who did not receive any anticoagulation in early 2020, the median time from the first symptom to dyspnea, hospital admission, and acute respiratory distress syndrome onset was 5, 7, and 8 days, respectively.^39^ This disease course indicates that there is only a narrow time window to initiate treatment and effectively prevent clinical deterioration. The delayed time to patient inclusion in the included trials may have precluded a therapeutic benefit, as microcirculatory thrombosis might have already taken place. This prompts the question, whether even earlier initiation of anticoagulation might be indicated for timely thromboembolic prophylaxis, if the time from symptom onset to initiation of treatment does not exceed a certain, yet undefined, threshold.

Noncritically ill patients with COVID‐19 receiving higher‐dose anticoagulation showed a numerical risk reduction of death compared with prophylactic‐dose anticoagulation, however, without reaching statistical significance. This contrasts with the findings in critically ill patients, who showed a similar risk of death between higher‐dose and prophylactic‐dose anticoagulation. Disease progression may be too advanced in critically ill patients to benefit from higher‐dose anticoagulation.^40^ Consequently, the use of higher‐dose anticoagulation might be considered, if at all, rather in noncritically ill patients hospitalized with COVID‐19, whose early disease state may still allow therapeutic benefit.^41^ However, there is currently insufficient evidence of survival benefit of higher‐dose anticoagulation in noncritically ill patients.

Elevated levels of d‐dimer may be associated with increased mortality and risk of complications in COVID‐19,^42^ although this remains controversial.^43^ It has been hypothesized that high d‐dimer levels may identify patients who could benefit from higher‐dose anticoagulation. In patients with markedly increased d‐dimer levels, higher‐dose anticoagulation was not associated with a lower risk of death when compared with prophylactic‐dose anticoagulation. Further clinical data and better mechanistic understanding are needed to definitively assess the therapy‐guiding potential of d‐dimer in COVID‐19.

The ATTACC trial was the largest trial (*n* = 2,219) and showed an increased probability of survival to hospital discharge with reduced use of cardiovascular or respiratory organ support with therapeutic‐dose vs. prophylactic‐dose anticoagulation. In‐hospital deaths alone showed no statistically significant difference between the two groups. Importantly, the timing of initiation of organ support, despite being supported by guidelines, ultimately remains a subjective decision by the treating physicians, who were aware of the anticoagulation treatment allocation in the ATTACC trial. The potential introduction of bias by subjective end points has already been addressed in the context of other open‐label trials on COVID‐19.^44^

Upcoming data from clinical trials investigating anticoagulation dosing strategies in patients hospitalized with COVID‐19 are eagerly awaited (FREEDOM COVID, NCT04512079; and ANTICOVID, NCT04808882).^45^ These emerging trial results may help to further define the potential clinical role of higher‐dose anticoagulation.

### Limitations

Our meta‐analysis has several limitations. First, its limited sample size precludes universal statements, especially in the subgroup analyses. Second, the included studies comprised critically ill and noncritically ill patients with partly varying definitions. Third, three different bleeding definitions were used, potentially skewing the major bleeding analysis. Fourth, one study also used the direct oral anticoagulant rivaroxaban in its therapeutic anticoagulation treatment arm. Fifth, the included trials were heterogeneous as primary outcomes, and, to a certain degree, follow‐up periods varied. Sixth, except for one, all trials had an open‐label design, which may have introduced bias in the ascertainment of thrombotic and bleeding events. Seventh, we included one study in our analysis that did not yet undergo the peer‐review process.^30^ However, sensitivity analyses confirmed that this trial had no significant impact on our main findings.

### Clinical implications and conclusion

Higher‐dose anticoagulation, mostly performed using LMWH, significantly reduced the risk of venous thromboembolic events, at the expense of a significantly increased risk of major bleeding. However, higher‐dose anticoagulation had no effect on all‐cause death. Consequently, this meta‐analysis does not support the routine use of therapeutic‐dose anticoagulation in patients hospitalized with COVID‐19. Therapeutic‐dose anticoagulation might improve outcomes in selected patients. In noncritically ill patients, we found a numerically lower risk of death with higher‐dose anticoagulation than with prophylactic‐dose anticoagulation, but without reaching statistical significance. Future studies may investigate whether early initiation of higher‐dose anticoagulation in noncritically ill patients is associated with improved outcomes. As of now, there is insufficient evidence of survival benefit of higher‐dose anticoagulation compared with prophylactic‐dose anticoagulation in noncritically ill and in critically ill patients.

## FUNDING

This research was not funded. G.G. is supported by a grant from the Austrian Science Funds (SFB54‐P04) and by the Federal Ministry of Education, Science and Research for performing the ACOVACT trial.

## CONFLICT OF INTEREST

The authors declared no competing interests for this work.

## AUTHOR CONTRIBUTIONS

A.J. and G.G. wrote the manuscript. G.G. designed the research. A.J. and G.G. performed the research. A.J., J.M.S.M., M.Z., B.J., and G.G. analyzed the data.

## Supporting information

Supplementary Material
