# Supplementary material for: Anticoagulant Treatment Regimens in Patients With Covid‐19: A Meta‐Analysis
Source: Clin Pharmacol Ther. 2021 Dec 19;111(3):614–23. doi: 10.1002/cpt.2504 (PMC9015466; doi:10.1002/cpt.2504)
Supplement: Supplementary file 1 — Supplementary Material [file CPT-111-614-s001.docx]

Supplemental Materials

**Anticoagulant treatment regimens in**

**patients with Covid-19: a meta-analysis**

Anselm Jorda, Jolanta M. Siller-Matula, Markus Zeitlinger,

Bernd Jilma, Georg Gelbenegger

**TABLE OF CONTENT**

[**Table S1** PRISMA 2020 Checklist 3](#_Toc88831575)

[**Table S2** Revised Cochrane risk-of-bias assessment of included trials 5](#_Toc88831576)

[**Table S3** Comprehensive overview of included trials 6](#_Toc88831577)

[**Table S4** Demographics of included trials 8](#_Toc88831578)

[**Figure S1 A-F** Funnel plots depicting the effects estimates of included trials 9](#_Toc88831579)

[**Figure S2** PRISMA flow diagram of study screening and selection (latest search on November 24^th^, 2021). 10](#_Toc88831580)

[**Figure S3** Forest plot depicting the pooled risk ratio of thromboembolic events between higher-dose and prophylactic-dose anticoagulation. 11](#_Toc88831581)

[**Figure S4** Forest plot depicting the risk ratio of pulmonary embolisms between higher-dose and prophylactic-dose anticoagulation. 11](#_Toc88831582)

[**Figure S5** Forest plot depicting the pooled risk ratio of strokes between higher-dose and prophylactic-dose anticoagulation. 12](#_Toc88831583)

[**Figure S6** Forest plot depicting the pooled risk ratio of myocardial infarctions between higher-dose and prophylactic-dose anticoagulation. 12](#_Toc88831584)

[**Figure S7** Forest plot depicting the pooled risk ratio of peripheral arterial thromboembolisms between higher-dose and prophylactic-dose anticoagulation. 13](#_Toc88831585)

[**Figure S8** Forest plot depicting the pooled risk ratio of any bleedings between higher-dose and prophylactic-dose anticoagulation. 13](#_Toc88831586)

[**Figure S9** Forest plot depicting the pooled risk ratio of thromboembolic events in non-critically and critically ill patients. 14](#_Toc88831587)

[**Figure S10** Forest plot depicting the pooled risk ratio of major bleeding events in non-critically and critically ill patients. 14](#_Toc88831588)

[**Figure S11** Forest plot depicting the pooled risk ratio of death between therapeutic-dose and prophylactic-dose anticoagulation. 15](#_Toc88831589)

[**Figure S12** Forest plot depicting the pooled risk ratio of thromboembolic events between therapeutic-dose and prophylactic-dose anticoagulation. 15](#_Toc88831590)

[**Figure S13** Forest plot depicting the pooled risk ratio of major bleeding events between therapeutic-dose and prophylactic-dose anticoagulation. 16](#_Toc88831591)

[**Figure S14** Forest plot depicting the pooled risk ratio of death between intermediate-dose and prophylactic-dose anticoagulation. 16](#_Toc88831592)

[**Figure S15** Forest plot depicting the pooled risk ratio of thromboembolic events between intermediate-dose and prophylactic-dose anticoagulation. 17](#_Toc88831593)

[**Figure S16** Forest plot depicting the pooled risk ratio of major bleeding events between intermediate-dose and prophylactic-dose anticoagulation. 17](#_Toc88831594)

[**Figure S17** Sensitivity analysis: Forest plot depicting the pooled risk ratio of death in the subgroup of non-critically ill patients without the ACTION trial. 17](#_Toc88831595)

# **Table S1** PRISMA 2020 Checklist

| **Section and Topic** | **Item #** | **Checklist item** | **Location where item is reported** |
| --- | --- | --- | --- |
| **TITLE** | | |  |
| Title | 1 | Identify the report as a systematic review. | Main text |
| **ABSTRACT** | | |  |
| Abstract | 2 | See the PRISMA 2020 for Abstracts checklist. | Main text |
| **INTRODUCTION** | | |  |
| Rationale | 3 | Describe the rationale for the review in the context of existing knowledge. | Main text |
| Objectives | 4 | Provide an explicit statement of the objective(s) or question(s) the review addresses. | Main text |
| **METHODS** | | |  |
| Eligibility criteria | 5 | Specify the inclusion and exclusion criteria for the review and how studies were grouped for the syntheses. | Main text |
| Information sources | 6 | Specify all databases, registers, websites, organisations, reference lists and other sources searched or consulted to identify studies. Specify the date when each source was last searched or consulted. | Main text |
| Search strategy | 7 | Present the full search strategies for all databases, registers and websites, including any filters and limits used. | Main text |
| Selection process | 8 | Specify the methods used to decide whether a study met the inclusion criteria of the review, including how many reviewers screened each record and each report retrieved, whether they worked independently, and if applicable, details of automation tools used in the process. | Main text |
| Data collection process | 9 | Specify the methods used to collect data from reports, including how many reviewers collected data from each report, whether they worked independently, any processes for obtaining or confirming data from study investigators, and if applicable, details of automation tools used in the process. | Main text |
| Data items | 10a | List and define all outcomes for which data were sought. Specify whether all results that were compatible with each outcome domain in each study were sought (e.g. for all measures, time points, analyses), and if not, the methods used to decide which results to collect. | Main text |
|  | 10b | List and define all other variables for which data were sought (e.g. participant and intervention characteristics, funding sources). Describe any assumptions made about any missing or unclear information. | Main text |
| Study risk of bias assessment | 11 | Specify the methods used to assess risk of bias in the included studies, including details of the tool(s) used, how many reviewers assessed each study and whether they worked independently, and if applicable, details of automation tools used in the process. | Main text |
| Effect measures | 12 | Specify for each outcome the effect measure(s) (e.g. risk ratio, mean difference) used in the synthesis or presentation of results. | Main text |
| Synthesis methods | 13a | Describe the processes used to decide which studies were eligible for each synthesis (e.g. tabulating the study intervention characteristics and comparing against the planned groups for each synthesis (item #5)). | Main text |
|  | 13b | Describe any methods required to prepare the data for presentation or synthesis, such as handling of missing summary statistics, or data conversions. | Main text |
|  | 13c | Describe any methods used to tabulate or visually display results of individual studies and syntheses. | Main text |
|  | 13d | Describe any methods used to synthesize results and provide a rationale for the choice(s). If meta-analysis was performed, describe the model(s), method(s) to identify the presence and extent of statistical heterogeneity, and software package(s) used. | Main text |
|  | 13e | Describe any methods used to explore possible causes of heterogeneity among study results (e.g. subgroup analysis, meta-regression). | Main text |
|  | 13f | Describe any sensitivity analyses conducted to assess robustness of the synthesized results. | Main text |
| Reporting bias assessment | 14 | Describe any methods used to assess risk of bias due to missing results in a synthesis (arising from reporting biases). | Main text |
| Certainty assessment | 15 | Describe any methods used to assess certainty (or confidence) in the body of evidence for an outcome. | Main text |
| **RESULTS** | | |  |
| Study selection | 16a | Describe the results of the search and selection process, from the number of records identified in the search to the number of studies included in the review, ideally using a flow diagram. | Main text |
|  | 16b | Cite studies that might appear to meet the inclusion criteria, but which were excluded, and explain why they were excluded. | Main text |
| Study characteristics | 17 | Cite each included study and present its characteristics. | Main text, Table 1 |
| Risk of bias in studies | 18 | Present assessments of risk of bias for each included study. | Main text, supplement |
| Results of individual studies | 19 | For all outcomes, present, for each study: (a) summary statistics for each group (where appropriate) and (b) an effect estimate and its precision (e.g. confidence/credible interval), ideally using structured tables or plots. | Main text, Table 1, supplement |
| Results of syntheses | 20a | For each synthesis, briefly summarise the characteristics and risk of bias among contributing studies. | Main text, supplement |
|  | 20b | Present results of all statistical syntheses conducted. If meta-analysis was done, present for each the summary estimate and its precision (e.g. confidence/credible interval) and measures of statistical heterogeneity. If comparing groups, describe the direction of the effect. | Main text |
|  | 20c | Present results of all investigations of possible causes of heterogeneity among study results. | n.a. |
|  | 20d | Present results of all sensitivity analyses conducted to assess the robustness of the synthesized results. | Main text |
| Reporting biases | 21 | Present assessments of risk of bias due to missing results (arising from reporting biases) for each synthesis assessed. | Supplement |
| Certainty of evidence | 22 | Present assessments of certainty (or confidence) in the body of evidence for each outcome assessed. | Supplement |
| **DISCUSSION** | | |  |
| Discussion | 23a | Provide a general interpretation of the results in the context of other evidence. | Main text |
|  | 23b | Discuss any limitations of the evidence included in the review. | Main text |
|  | 23c | Discuss any limitations of the review processes used. | Main text |
|  | 23d | Discuss implications of the results for practice, policy, and future research. | Main text |
| **OTHER INFORMATION** | | |  |
| Registration and protocol | 24a | Provide registration information for the review, including register name and registration number, or state that the review was not registered. | Main text |
|  | 24b | Indicate where the review protocol can be accessed, or state that a protocol was not prepared. | Main text |
|  | 24c | Describe and explain any amendments to information provided at registration or in the protocol. | Main text |
| Support | 25 | Describe sources of financial or non-financial support for the review, and the role of the funders or sponsors in the review. | Main text |
| Competing interests | 26 | Declare any competing interests of review authors. | Main text |
| Availability of data, code and other materials | 27 | Report which of the following are publicly available and where they can be found: template data collection forms; data extracted from included studies; data used for all analyses; analytic code; any other materials used in the review. | Main text |

# **Table S2** Revised Cochrane risk-of-bias assessment of included trials

| **Study** | Domain 1: Risk of bias arising from the randomization process | Domain 2: Risk of bias due to deviations from the intended interventions (*effect of assignment to intervention*) | Domain 2: Risk of bias due to deviations from the intended interventions (*effect of adhering to intervention*) | Domain 3: Risk of bias due to missing outcome data | Domain 4: Risk of bias in measurement of the outcome | Domain 5: Risk of bias in selection of the reported result | | **Overall risk of bias** |
| --- | --- | --- | --- | --- | --- | --- | --- | --- |
| **ACTION, 2021** | Low risk | Low risk | Low risk | Low risk | Some concern | | Low risk | **Some concern** |
| **ATTAC, 2021** | Low risk | Low risk | High risk | Low risk | Some concern | | Low risk | **High risk** |
| **BEMICOP, 2021** | Low risk | Low risk | Low risk | Low risk | Some concern | | Low risk | **Some concern** |
| **HEPCOVID, 2021** | Low risk | Low risk | Low risk | Low risk | Low risk | | Low risk | **Low risk** |
| **HESACOVID, 2020** | Low risk | Low risk | Some concerns | Low risk | Some concern | | Low risk | **Some concerns** |
| **INSPIRATION, 2021** | Low risk | Low risk | Low risk | Low risk | Some concern | | Low risk | **Some concern** |
| **Perepu et al., 2021** | Low risk | Low risk | Low risk | Low risk | Low risk | | Low risk | **Low risk** |
| **RAPID,**  **2021** | Low risk | Low risk | Low risk | Low risk | Some concern | | Low risk | **Some concern** |
| **REMAP-CAP, 2021** | Low risk | Low risk | High risk | Low risk | Some concern | | Low risk | **High risk** |
| **XCOVID-19,**  **2021** | Low risk | Low risk | Low risk | Low risk | Some concern | | Low risk | **Some concern** |

# **Table S3** Comprehensive overview of included trials

| **Study** | **Study Design** | **Treatment** | **Study Population** | **Inclusion criteria** | **Exclusion criteria** | **Primary Efficacy Endpoint** | **Secondary Efficacy Endpoint** | **Safety Endpoint** |
| --- | --- | --- | --- | --- | --- | --- | --- | --- |
| **ACTION, 2021** | open-label (with blinded adjudication), multicenter, pragmatic RCT | Therapeutic: 1 mg/kg twice daily OR rivaroxaban 20 mg twice daily  versus  Prophylactic: 40 mg once daily | hospitalised with Covid-19 (stable and unstable) | -patients with confirmed diagnosis of COVID-19 admitted to hospital  -duration of symptoms related to hospitalisation ≤14 days  -patients ≥ 18 years old  -d-dimer above the ULN  -agreement to participate by providing the informed consent form | -patients with indication for therapeutic anticoagulation during inclusion (e.g, diagnosis of VTE, AF, mechanical valve prosthesis)  -platelets <50,000 /mm^3^  -use of aspirin >100 mg  -use of P2Y12 inhibitor (clopidogrel, prasugrel, ticagrelor)  -chronic use of NSAIDs  -sustained uncontrolled systolic BP ≥180 mm Hg or diastolic BP ≥100 mm Hg  -INR >1·5  -patients contraindicated to therapeutic anticoagulation (active bleeding, liver failure, blood dyscrasia or prohibitive haemorrhage risk as  evaluated by the investigator)  -Patients with DIC  -history of haemorrhagic stroke or any intracranial bleeding at any time in the past or current intracranial neoplasm (benign or malignant), cerebral metastases, arteriovenous malformation, or aneurysm  -active cancer (excluding non-melanoma skin cancer) defined as cancer not in remission or requiring active chemotherapy or adjunctive therapies such as immunotherapy or radiotherapy  -hypersensitivity to rivaroxaban  -use of strong inhibitors of cytochrome P450 (CYP) 3A4 and/or P-gp (e.g., protease inhibitors, ketoconazole, itraconazole) and/or use of P-gp  and strong CYP3A4 inducers (including, but not limited to, rifampin/rifampicin, rifabutin, rifapentine, phenytoin, phenobarbital, carbamazepine, or St. John's Wort)  -known HIV infection  -creatinine clearance <30 mL/min  -pregnancy or breastfeeding | hierarchical composite of time to death, duration of hospitalisation, or duration of supplemental oxygen use through 30 days | composite outcomes of venous thromboembolism, acute myocardial infarction, any stroke (ischaemic or haemorrhagic), systemic embolism, and major adverse limb events, with and without all-cause death | major or clinically relevant non-major bleeding |
| **ATTAC, 2021** | open-label, multicenter, adaptive, multiplatform RCT | Therapeutic: 1 mg/kg twice daily  versus  Prophylactic: 40 mg once daily | hospitalized with Covid-19 (non-critically ill) | -≥18a  -expected hospitalization of at ≥48 to 72 hours  -SARS-CoV-2 infection  -less than 48 hours form ICU admission | -platelet count <50x 10^9^/L  -hemoglobin <8 g/dL  -heparin-induced thrombocytopenia  -dual antiplatelet therapy  -mechanical ventilation  -poor prognosis  -increased bleeding risk/contraindication to anticoagulation | organ support–free days, evaluated on an ordinal scale that combined in-hospital death and the number of days free of cardiovascular or respiratory organ support up to day 21 | -survival until hospital discharge  -survival without receipt of organ support  -survival without receipt of invasive mechanical ventilation  -survival without mechanical respiratory support  -length of hospital stay  -major thrombotic event or death (a composite of myocardial infarction, pulmonary embolism, ischemic stroke, systemic arterial embolism, or in-hospital death)  -thrombotic event including deep venous thrombosis | -major bleeding (according to ISTH)  -laboratory-confirmed HIT |
| **BEMICOP,**  **2021** | open-label, multicenter, randomized, controlled trial | Therapeutic: 115 IU/kg once daily (bemiparin)  versus  Prophylactic: 3500 IU once daily (bemiparin) | admitted to conventional ward with Covid-19 | -Age ≥ 18 years-old  -Hospitalization at the conventional ward due to mild or moderate (CURB65 ≥ 2 points and Sat.O2>90%) COVID-19 pneumonia  -Maximum allowed time between hospitalization and randomization is 48 hours  -3-4 points according to the WHO ordinal scale  -Confirmed COVID-19 diagnosis by PCR or other validated test.  -Baseline D-Dimer >500 ng/mL  -Signed informed consent  -The patient, according to investigator’s opinion, is able to deal with all the requirements of the clinical trial | -Need of intensive care unit admission  -Moderate or severe adult respiratory distress syndrome  -Body weight <50Kg  -Creatinine clearance (Cockroft-Gault) <30ml/min  -Severe liver disease (elevation of hepatic enzymes 3 times above the upper limit of normal)  -Thrombocytopenia <75,000/mm3  -History of coagulopathy or thrombocytopathy  -Active bleeding of increased bleeding risk due to impairment of haemostasis  -Recent (1 month) central nervous system o gastrointestinal bleeding  -Lesions of surgery involving central nervous system, eyes or inner ear in the last 2 months  -Presence of organic lesions with high bleeding risk (e.g. active peptic ulcer, hemorragic stroke, brain aneurism o tumor)  -Planned surgery or interventional procedure requiring regional anesthesia  -Uncontrolled arterial hypertension  -Acute or subacute bacterial endocarditis  -Need of therapeutic anticoagulation for other reasons (e.g. atrial fibrillation, valvular prosthesis, venous thromboembolism)  -Need of antiplatelet therapy  -Simultaneous participation in another clinical trial (use of drugs against COVID-19 in the setting of local clinical management protocols is allowed)  -Previous history of heparin-induced thrombocytopenia  -Hypersensitivity or allergy to sodic bemiparin, heparin, compounds of porcine origin or any of the excipients | -composite outcome that includes death, ICU admission, need of invasive or non-invasive mechanical ventilation support  -development of moderate or severe acute respiratory distress syndrome (according to Berlin criteria)  -venous or arterial thrombosis at day 10 | -need of ICU or mechanical ventilation at 10 days and at 30 days  -Death at 10 days and 30 days  -Objectively diagnosed venous or arterial thromboembolism at 10 days and 30 days  -% of patients with negative SARS-CoV-2 PCR at 10 days  -% of patients discharged at day 10  -% of patients with clinical improvement according to the Brescia-COVID score at 10 days  -% of patients with blood IL-6 levels more than 2 times above the upper limit of normal  -% of patents with D-dimer > 1 ug/mL at 5 and 10 days  -% of patients with SIC score ≥ 4 points at 5 and 10 days  -% of patients with radiological improvement at 5 and 10 days | -Major bleeding (ISTH definition) at 10 days -Clinically relevant non-major bleeding (ISTH definition) at 10 days  - Treatment-related adverse events - Need of study-drug interruption due to adverse event |
| **HEP-COVID, 2021** | multicenter, active control randomized clinical trial | Therapeutic: 1 mg/kg twice daily  versus  Prophylactic: 30-40 mg once daily | hospitalized with Covid-19  and elevated d-dimer  (ICU and non-ICU patients) | -Subject (or legally authorized representative) provides written informed consent prior to initiation of any study procedures.  -Understands and agrees to comply with planned study procedures.  -Male or non-pregnant female adult ≥18 years of age at time of enrollment.  -Subject consents to randomization within 72 hours of hospital admission or transfer from  another facility within 72 hours of index presentation.  -Subjects with a positive COVID-19 diagnosis by nasal swab or serologic testing  -Hospitalized with a requirement for supplemental oxygen  -Have:  --Either a D- Dimer > 4.0 X ULN OR  --Sepsis-induced coagulopathy (SIC) score of ≥4 | -Indications for therapeutic anticoagulation  -Absolute contraindication to anticoagulation including:  --active bleeding,  --recent (within 1 month) history of bleed,  --dual (but not single) antiplatelet therapy,  --active gastrointestinal and intracranial cancer,  --history of bronchiectasis or pulmonary cavitation,  --Hepatic failure with a baseline INR > 1.5,  --CrCl < 15ml/min,  --a platelet count < 25,000,  --a history of heparin-induced thrombocytopenia (HIT) within the past 100 days or in the presence of circulating antibodies.  --contraindications to enoxaparin including a hypersensitivity to enoxaparin sodium, hypersensitivity to heparin or pork products, hypersensitivity to benzyl alcohol  --pregnant females  --inability to give or designate to give informed consent  --participation in another blinded trial of investigational drug therapy for COVID-19 | -VTE (symptomatic upper or lower extremity deep vein thrombosis, asymptomatic lower extremity proximal deep vein thrombosis, symptomatic pulmonary embolism, splanchnic vein thrombosis, or cerebral sinus thrombosis)  -ATE (myocardial infarction, ischemic stroke, peripheral or systemic ATE)  -death from any cause within 30 ± 2 days after randomization | -composite primary outcome within 14 days after admission  -progression to acute respiratory distress syndrome  -new-onset atrial fibrillation  -acute kidney injury  -non-fatal cardiac arrest  -endotracheal intubation  -extracorporeal membrane oxygenation  -rehospitalization within 30 ± 2 days | major bleeding based on International Society on Thrombosis and Haemostasis criteria within 30 ± 2 days |
| **HESACOVID, 2020** | open-label, single-center, phase II RCT | Therapeutic: 1 mg/kg twice daily  versus  Prophylactic: 40 mg once daily | hospitalized with Covid-19 (and ARDS) | -≥18a  -confirmed SARS-CoV-2 infection  -presence of ARDS according to Berlin definition  -severe clinical presentation with respiratory failure requiring mechanical ventilation  -D-dimer levels greater than 1000 μg/L  -prothrombin time/international normalized ratio (INR) < 1.5  -activated partial thromboplastin time/ratio < 1.5  -platelet count greater than 100,000/mm^3^ | -over 85a  -creatinine clearance < 10 mL/min  -severe circulatory shock  -chronic renal failure in renal replacement therapy  -Child B and C chronic liver disease  -advanced diseases, such as active cancer, heart failure with functional class III and IV (New York Heart Failure Association), chronic obstructive pulmonary disease using home oxygen, advanced dementia, significant disability from stroke or severe head injury, cardiorespiratory arrest  -pregnant women  -recent major surgery or severe trauma in the last 3 weeks  -recent stroke in the last 3 months  -active bleeding  -blood dyscrasia such as hemophilia, Von Willebrand factor deficiency, participation in another clinical investigation  -indication for therapeutic anticoagulation due to pulmonary embolism  -acute coronary syndrome | variation in gas exchange over time evaluated through the ratio of partial pressure of arterial oxygen (PaO2) to the fraction of inspired oxygen (FiO2) at baseline, 7, and 14 days after randomization | -time until successful liberation from mechanical ventilation  -ventilator-free days (during the 28 days after inclusion in the study  -numbers of days without mechanical ventilation  -the variation in D-dimer levels collected at baseline during inclusion in the study and repeated 72–96 h later  -all- cause 28-day mortality, in-hospital mortality  -ICU-free days at 28 days | -bleeding |
| **INSPIRATION, 2021** | open-label, multicenter RCT | Intermediate: 1 mg/kg once daily  versus  Prophylactic: 40 mg once daily | admitted to ICU with Covid-19 | -adult patients (≥18 years), with PCR-confirmed COVID-19 admitted to ICU within 7 days of initial hospitalization, who do not have another firm indication for anticoagulation (such as mechanical valve, high-risk AF, VTE, or left ventricular thrombus)  -estimated survival of at least 24 hours at the discretion of enrolling physician | -weight <40Kg  -use of systemic anticoagulation for another indication (mechanical valve, ECMO, AF, left ventricular thrombus, or diagnosed VTE)  -overt bleeding at the day of enrollment  -known major bleeding within 30 days (according to the Bleeding Academic Research  Consortium (BARC) definition1)  -platelet count <50,000/Fl  -pregnancy (as confirmed by beta-HCG testing among female patients <50 years)  -history of heparin induced thrombocytopenia or immune thrombocytopenia  -ischemic stroke within the past 2 weeks  -major head or spinal trauma in the past 30 days  -craniotomy/major neurosurgery within the past 3 months  -known brain metastases or vascular malformations (aneurysm)  -presence of an epidural, spinal or pericardial catheter  -major surgery other than neurosurgery within 14 days prior to enrollment  -coexistence of severe obesity (weight >120Kg or BMI>35Kg/M2 along with severe renal insufficiency defined as CrCl<30 mL/min)  -allergic reaction to study medications  -lack or withdrawal of informed consent | composite of adjudicated acute VTE, arterial thrombosis, treatment with extracorporeal membrane oxygenation (ECMO), or all-cause mortality within 30 days | -all-cause mortality  -adjudicated VTE  -ventilator-free days | - major bleeding (Bleeding Academic Research Consortium type 3 or 5)  -severe thrombocytopenia (platelet count <20 ×103/μL) |
| **Perepu et al., 2021** | open-label, multi-center RCT | Intermediate: 1 mg/kg once daily  versus  Prophylactic: 40 mg once daily | hospitalized with Covid-19 (ICU and/or coagulopathy) | -laboratory confirmed SARS-CoV-2 infection  -age ≥18 years  -requires hospital admission for further clinical management  -modified ISTH Overt DIC score ≥ 3 OR any Modified ISTH Overt DIC Score and admitted to an intensive care unit | -indication for full therapeutic-dose anticoagulation  -acute venous thromboembolism (deep vein thrombosis or pulmonary  embolism) within prior 3 months  -acute cardiovascular event within prior 3 months  -acute stroke (ischemic or hemorrhagic) within prior 3 months  -active major bleeding  -severe thrombocytopenia (<25,000/mm^3^)  -increased risk of bleeding, as assessed by the investigator  -acute or chronic renal insufficiency with estimated Creatinine Clearance< 30  ml/min calculated by the modified Cockcroft and Gault formula  -weight < 40 kg  -known allergies to ingredients contained in enoxaparin or allergy to heparin  products such as history of heparin induced thrombocytopenia  -current pregnancy | all-cause mortality at 30 days | -acute kidney injury, defined as estimated creatinine clearance <30 ml/min  -arterial or venous thrombosis confirmed with imaging  -major bleeding  -minor bleeding | n.a. |
| **RAPID,**  **2021** | parallel, pragmatic, adaptive multi-center, open-label RCT | Therapeutic: 1 mg/kg twice daily  versus  Prophylactic: 40 mg once daily | hospitalized with Covid-19  (and elevated d-dimer) | -laboratory confirmed COVID-19  -positive test prior to hospital admission OR within first 5 days (i.e. 120 hours) after hospital admission  -admitted to hospital for COVID-19  -one D-dimer value above ULN (within 5 days (i.e. 120 hours) of hospital admission) AND EITHER: D-Dimer ≥2 times ULN OR D-Dimer above ULN and Oxygen saturation ≤ 93% on room air  -over 18 years of age  -informed consent from the patient (or legally authorized substitute decision maker) | -pregnancy  -hemoglobin <80 g/L in the last 72 hours  -platelet count <50 x 10^9^/L in the last 72 hours  -known fibrinogen <1.5 g/L (if testing deemed clinically indicated by the treating physician prior to the initiation of anticoagulation)  -known INR >1.8 (if testing deemed clinically indicated by the treating physician prior to the initiation of anticoagulation)  -patient already on intermediate dosing of LMWH that cannot be changed (determination of what constitutes an intermediate dose is to be at the discretion of the treating clinician taking the local institutional thromboprophylaxis protocol for high risk patients into consideration)  -patient already on therapeutic anticoagulation at the time of screening (low or high dose nomogram UFH, LMWH, warfarin, direct oral anticoagulant (any dose of dabigatran, apixaban, rivaroxaban, edoxaban)  -patient on dual antiplatelet therapy, when one of the agents cannot be stopped safely  -known bleeding within the last 30 days requiring emergency room presentation or hospitalization  -known history of a bleeding disorder of an inherited or active acquired bleeding disorder  -known history of heparin-induced thrombocytopenia  -known allergy to UFH or LMWH  -admitted to the intensive care unit at the time of screening  -treated with non-invasive positive pressure ventilation or invasive mechanical ventilation at the time of screening (of note: high flow oxygen delivery via nasal cannula is acceptable and is not an exclusion criterion)  -imminent death according to the judgement of the most responsible physician  -enrollment in another clinical trial of antithrombotic therapy involving pre-intensive care unit hospitalized patients | composite of ICU admission, non-invasive (bilevel or continuous positive airway pressure) or invasive mechanical ventilation, or death up to 28 days | -all-cause death  -composite of any mechanical ventilation or all-cause death  -ICU admission or all-cause death  -ventilator-free days alive  -organ support-free days alive  -ICU-free days alive  -hospital-free days alive  -renal replacement therapy  -venous thromboembolism  -arterial thromboembolism  -D-dimer level at 2 days ± 24 hours post-randomization | -major bleeding as defined by the International Society on Thrombosis and Haemostasis  -red blood cell transfusion (≥1 unit)  -transfusion of hemostatic blood components or products  -heparin-induced thrombocytopenia |
| **REMAP-CAP, 2021** | open-label, multicenter, adaptive, multiplatform, RCT | Therapeutic: 1 mg/kg twice daily  versus  Prophylactic: 40 mg once daily | hospitalized with Covid-19 (critically ill) | -≥18a  -expected hospitalization of at ≥48 to 72 hours  -SARS-CoV-2 infection  -less than 48 hours form ICU admission | -platelet count <50x 10^9^/L  -hemoglobin <8 g/dL  -heparin-induced thrombocytopenia  -dual antiplatelet therapy  -mechanical ventilation  -poor prognosis  -increased bleeding risk/contraindication to anticoagulation | organ support–free days, evaluated on an ordinal scale indicating the number of days free of cardiovascular or respiratory organ support up to day 21 | -survival to hospital discharge  -major thrombotic events or death (a composite of myocardial infarction, pulmonary embolism, ischemic stroke, systemic arterial embolism, or in-hospital death)  -any thrombotic events (major thrombotic events or deep-vein thrombosis)  -death | -major bleeding  -laboratory-confirmed heparin-induced thrombocytopenia |
| **XCOVID-19, 2021** | multicenter, open-label, randomized trial | Intermediate: 40 mg twice daily  versus  Prophylactic: 40 mg once daily | Admitted to general wards with COVID-19 | -aged >=18 years  -admitted to hospital with laboratory-confirmed SARS-CoV- 2 infection | -patients admitted directly to intensive care unit  -patients with estimated creatinine clearance <15 ml/min/1.73m2.  -patients needing anticoagulant for prior indication  -patients at high bleeding risk or experiencing clinically significant bleeding  -patients involved in clinical trial protocols restricting patients from concurrent studies  -patients with any other significant disease or disorder which, in the opinion of the Investigator,  may either put the participants at risk because of participation in the trial, or may influence the result of the trial, or the participant’s ability to participate in the trial | incidence of VTE (a composite of asymptomatic or symptomatic proximal  DVT diagnosed by serial CUS, and symptomatic PE diagnosed by CTA) | -major adverse events (composite of overall death, VTE, use of mechanical ventilation, stroke, acute myocardial infarction and admission to ICU)  -each single component of the primary endpoint  -maximum sequential organ failure assessment (SOFA)  -levels of C-reactive protein, D-dimer, IL-6 and hs-troponin  -Acute Respiratory Distress Syndrome (ARDS)  -length of hospital stay  -changes in right ventricular function at trans-thoracic echocardiography between admission and follow-up  -composite of death, stroke and myocardial infarction at 30 days | -major bleeding according to the International Society on Thrombosis and Haemostasis (ISTH) bleeding scale  -type 3 and 5 bleeding events according to the Bleeding Academic Research Consortium (BARC)  -heparin-induced thrombocytopenia (HIT)  -dyspnea according to the Borg scale. |

# **Table S4** Demographics of included trials

| **Study** | **Patients included** | **Median days from symptom onset to randomization** | **Mean age (years)** | **Male**  **(%)** | **Mean Body Mass Index** | **Critically ill (%)** | **Mechanical Ventilation (%)** |
| --- | --- | --- | --- | --- | --- | --- | --- |
| ACTION, 2021 | 615 (311 vs 304) | 10 | 57 | 60 | 30 | 6 | 6 |
| ATTAC, 2021 | 2219 (1171 vs 1048) | not reported | 60 | 59 | 30^a^ | 0 | 0 |
| BEMICOP,  2021 | 65  (32 vs 33) | 8 | 63 | 63 | 26 | 0 | 0 |
| HEP-COVID,  2021 | 253  (129 vs 124) | not reported | 67 | 68 | 31 | 33 | 5 |
| HESACOVID, 2020 | 20 (10 vs 10) | not reported | 57 | 80 | 34 | 100 | 100 |
| INSPIRATION, 2021 | 562 (276 vs 286) | 11 | 61 | 58 | 27 ^a^ | 100 | 20 |
| Perepu et al., 2021 | 173 (87 vs 86) | not reported | 64 | 56 | 31^a^ | 62 | 23 |
| RAPID,  2021 | 465  (228 vs 237) | 7^a^ | 60 | 57 | 30 | 0 | 0 |
| REMAP-CAP, 2021 | 1098 (534 vs 564) | not reported | 61 | 70 | 30^a^ | 100 | 29 |
| XCOVID-19, 2021 | 183  (91 vs 92) | 6-7 | 59 | 63 | 25 | 0 | 0 |

^a^mean

# **Figure S1 A-F** Funnel plots depicting the effects estimates of included trials

# **Figure S2** PRISMA flow diagram of study screening and selection (latest search on November 24^th^, 2021).

**Figure S3** Forest plot depicting the pooled risk ratio of thromboembolic events between higher-dose and prophylactic-dose anticoagulation.

The composite of thromboembolic events includes arterial thromboembolic events and venous thromboembolic events.

# **Figure S4** Forest plot depicting the risk ratio of pulmonary embolisms between higher-dose and prophylactic-dose anticoagulation.

The trial by Perepu et al. did not provide sufficient information to infer the number of pulmonary embolisms per group.

# **Figure S5** Forest plot depicting the pooled risk ratio of strokes between higher-dose and prophylactic-dose anticoagulation.

# **Figure S6** Forest plot depicting the pooled risk ratio of myocardial infarctions between higher-dose and prophylactic-dose anticoagulation.

# **Figure S7** Forest plot depicting the pooled risk ratio of peripheral arterial thromboembolisms between higher-dose and prophylactic-dose anticoagulation.

# **Figure S8** Forest plot depicting the pooled risk ratio of any bleedings between higher-dose and prophylactic-dose anticoagulation.

The definition of any bleedings includes major and non-major bleedings. Studies only reporting on major bleedings where not included in this analysis.

# **Figure S9** Forest plot depicting the pooled risk ratio of thromboembolic events in non-critically and critically ill patients.

The composite of thromboembolic events includes arterial thromboembolic events and venous thromboembolic events.

# **Figure S10** Forest plot depicting the pooled risk ratio of major bleeding events in non-critically and critically ill patients.

# **Figure S11** Forest plot depicting the pooled risk ratio of death between therapeutic-dose and prophylactic-dose anticoagulation.

# **Figure S12** Forest plot depicting the pooled risk ratio of thromboembolic events between therapeutic-dose and prophylactic-dose anticoagulation.

The composite of thromboembolic events includes arterial thromboembolic events and venous thromboembolic events.

# **Figure S13** Forest plot depicting the pooled risk ratio of major bleeding events between therapeutic-dose and prophylactic-dose anticoagulation.

# **Figure S14** Forest plot depicting the pooled risk ratio of death between intermediate-dose and prophylactic-dose anticoagulation.

# **Figure S15** Forest plot depicting the pooled risk ratio of thromboembolic events between intermediate-dose and prophylactic-dose anticoagulation.

The composite of thromboembolic events includes arterial thromboembolic events and venous thromboembolic events.

# **Figure S16** Forest plot depicting the pooled risk ratio of major bleeding events between intermediate-dose and prophylactic-dose anticoagulation.

# **Figure S17** Sensitivity analysis: Forest plot depicting the pooled risk ratio of death in the subgroup of non-critically ill patients without the ACTION trial.
